# Prevalence of Missed Canals and Their Association with Apical Periodontitis in Posterior Endodontically Treated Teeth: A CBCT Study

**DOI:** 10.1155/2021/9962429

**Published:** 2021-06-28

**Authors:** Mohammed Mashyakhy, Fatimah Ali Hadi, Hashimah Alhassan Alhazmi, Rawan Ali Alfaifi, Fatimah Saleem Alabsi, Hashim Bajawi, Mazen Alkahtany, Abdulaziz AbuMelha

**Affiliations:** ^1^Department of Restorative Dental Sciences, College of Dentistry, Jazan University, Jazan, Saudi Arabia; ^2^General Dental Practitioner, Jazan, Saudi Arabia; ^3^Department of Restorative Dental Sciences, College of Dentistry, King Saud University, Riyadh, Saudi Arabia; ^4^Department of Restorative Dental Sciences, College of Dentistry, King Khalid University, Abha, Saudi Arabia

## Abstract

**Background:**

This study aimed to assess the prevalence of missed canals in endodontically treated teeth and their association with apical periodontitis in a Saudi Arabian population using CBCT.

**Materials and Methods:**

A total of 208 CBCT radiographs were investigated. For each tooth, radiographs of axial, coronal, and sagittal segments were acquired to appraise the external as well as the internal structure of the root canal system and apical area. In root canal-treated teeth, unfilled canals appearing from the cementoenamel junction to the apex were defined as missed untreated canals; and a periapical lesion was considered when disruption of the lamina dura was detected, and the low-density area associated with the radiographic apex was at least twice the width of the periodontal ligament space. The data were presented as frequencies and percentages. The *Z*-test was used to analyze the differences in proportions with the significance level set at *P* value <0.05.

**Results:**

The overall prevalence of missed canals among endodontically treated teeth was 18%. The prevalence of missed canals was higher in maxillary first molars with 40.6%. The overall prevalence of apical periodontitis among teeth with missed canals was 90%. It was 84.2% in the maxilla and 100% in the mandible. The second mesiobuccal canal in the maxillary first molars and mesiobuccal and distobuccal canals in mandibular teeth were the most missed canals.

**Conclusion:**

Apical periodontitis in root canal-treated teeth with missed canals was high (90%), with most identified missed canals in maxillary and mandibular first molars.

## 1. Introduction

The principal objectives of root canal therapy (RCT) are to perform adequate biomechanical shaping, cleaning, and filling the entire root canal system (RCS) 3-dimensionally (3D). Inability to do so inevitably leads to an unfavourable outcome [1]. Studies have shown many factors attributed to endodontic failure, including but not limited to persistent bacterial infection [2], inadequate root filling [3], and untreated/missed canals [4]. Few studies reported RCT failure due to missed canals ranging from 12% to 42% in different populations [5–8].

The importance of locating all existing canals within the RCS to achieve optimal prognosis has been discussed by many authors, and potential negative effects of untreated canals on the treatment result have been debated with overwhelming evidence of missed canals in failed cases requiring endodontic retreatment [5, 9, 10]. Apical periodontitis (AP) was observed in 98% of the teeth with untreated canals, which was a significantly greater frequency in comparison with teeth with all canals treated [6]. Clinician's lack of knowledge of the RCS and its complexity may result in missing of canals during RCT. These canals serve as a pool of microorganisms, which is the main cause of forming or persistent apical periodontitis [11] and may have a negative impact on the prognosis [12, 13]. The conventional intraoral radiograph was used in old studies [14–17] to evaluate the posttreatment apical periodontitis, and because of its inherited limitations as a 2-dimensional (2D) method to assess a 3D object, locating missed treated canals was a challenge. Recently, cone-beam computed tomographic (CBCT) imaging allows for precise visualization of a given tooth in 3D [7]. The prevalence of missed canals in endodontically treated teeth and their association with apical lesions have been evaluated by means of CBCT in recent studies in different populations [6–8], where it showed more accurate and reliable results.

Upon literature search, no study was conducted in Saudi Arabia utilizing CBCT to evaluate the prevalence of missed canals and their association with AP. The need to do so is paramount to emphasize the importance of understanding the root canal anatomy before initiating RCT and to highlight the most affected teeth and manage them accordingly.

So, the aim of this study, therefore, was to assess the prevalence of missed canals in endodontically treated teeth and their association with apical periodontitis in a Saudi Arabian population utilizing *in vivo* CBCT.

## 2. Materials and Methods

This cross-sectional study included 208 CBCT images which were retrospectively collected from the archive of the College of Dentistry, Jazan University, Saudi Arabia, between January 2017 and July 2019. These CBCTs belong to patients (100 males and 108 females) referred to the College of Dentistry for dental treatment (no patient was exposed to radiography for the sake of the study; all CBCTs were taken for other diagnostic purposes). Ethical approval from the Standing Committee for Scientific Research Ethics at Jazan University was obtained before commencing the study (ref no.: REC41/1-008). All endodontically treated and nontreated posterior maxillary and mandibular teeth were included except third molars. Distorted/unclear images, retained primary teeth, or remaining roots on the CBCT radiographs were excluded. Initially, more than 250 full mouth CBCT scans were collected, and after applying the inclusion and exclusion criteria, only 208 scans were included in the study. The CBCT machine, 3D Accuitomo 170 (MORITA, Japan), was used with the following features: 90 Kv, 5–8 mA, 17.5 s exposure time, and 0.25 mm voxel size. The software imaging program MORITA i‐Dixel 3D was used for processing of the CBCT radiographs. Each scan was evaluated by 3 calibrated general practitioners as a group; when the group encountered a doubt, an expert endodontist in reading CBCT was consulted for the final decision.

For each tooth, radiographs of axial, coronal, and sagittal segments were acquired to appraise the external as well as the internal structure of the root canal system and apical area. For the purpose of this study, unfilled canals, appearing from the cementoenamel junction to the apex, were defined as missed untreated canals. AP was considered when disruption of the lamina dura was detected, and the low-density area associated with the radiographic apex was at least twice the width of the periodontal ligament space [18, 19].

### 2.1. Statistical Analysis

The collected data were analyzed using a statistical software program for Windows (SPSS V25; IBM, Chicago, IL). The results were presented as frequencies and percentages. The *Z*-test was used to analyze the differences in proportions with the significance level set at *P* value <0.05.

## 3. Results

**Figure 1:**
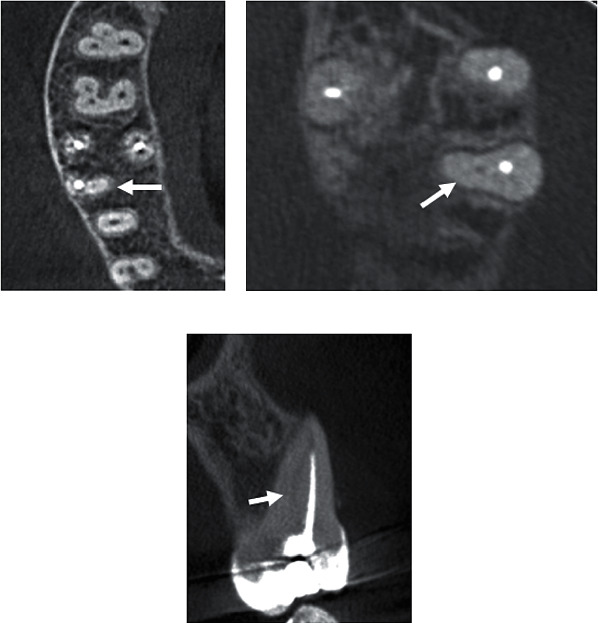
CBCT slices of the maxillary first molar: (a, b) axial section with the missed MB2 canal (arrows); (c) coronal section with the missed MB2 canal (arrow).

**Figure 2:**
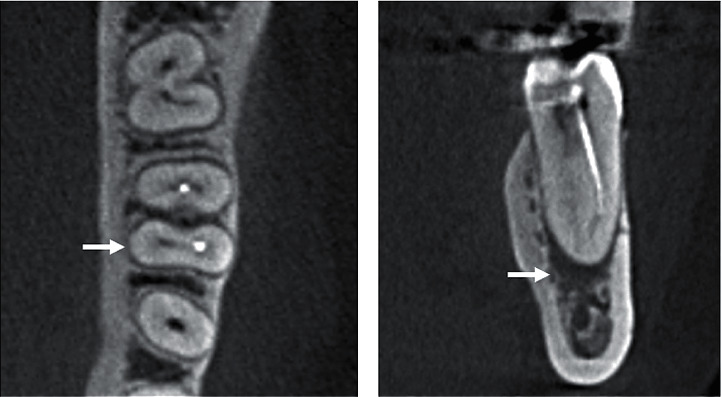
CBCT slices of the mandibular first molar: (a) axial section with the missed ML canal (arrow); (b) coronal section with the missed ML canal and the presence of apical radiolucency (arrow).

The prevalence of RCT among 3046 screened teeth (1529 maxillary and 1517 mandibular) was 5.4% (*n* = 165 teeth). The overall prevalence of missed canals among endodontically treated teeth was 18% (*n* = 30 out of 165). The prevalence in the maxilla was higher than in the mandible (19 compared to 11). The prevalence of missed canals was higher in maxillary first molars with 40.6% (CI_95%_ = 24%–58%; *P*=0.380) followed by maxillary first premolars with 13.6% (CI_95%_ = 0%–28%; *P* < 0.001), while the lowest prevalence was found in maxillary second premolars with 4.3% (CI_95%_ = 0%–13%; *P* < 0.001). All missed canals in the mandible were found in the first molars with a prevalence of 25% (CI_95%_ = 12%–38%; *P* < 0.001) (Table 1). Apical periodontitis was highly associated with missed canals. The overall prevalence of AP among teeth with missed canals was 90% (27 out of 30). It was 84.2% in the maxilla (Table 2) and 100% in the mandible (Table 3). The second mesiobuccal (MB2) canal in the maxillary first molars was tthe most missed treated canal (Figure 1), followed by the distobuccal canal. In the mandible, the mesiobuccal and distobuccal canals were the most missed canals, followed by mesiolingual and distolingual canals (Figure 2).

## 4. Discussion

*In vivo* CBCT scan was used in this study to evaluate the frequency of missed canals and their association with AP in RCT teeth. CBCT has many benefits that provides 3D images allowing the analysis of multiple slices per tooth [20], and it is less susceptible to errors in identifying RCS than conventional and digital periapical radiographs [5, 21]. In addition, comparative studies showed more accurate results of CBCT compared to conventional radiographs in detecting AP, especially in a small lesion with minimal apical bone destruction [22–25]. Thus, the inherited limitations of conventional radiography may negatively affect the detection of missed canals and AP in old studies, where it might show false negative results.

Endodontic literature has shown clearly that a high frequency of missed canals in RCT teeth needs nonsurgical endodontic retreatment. Hoen and Pink [5] found missed canals in 42% of all teeth that were retreated nonsurgically, while Karabucak et al. [7] reported the overall prevalence of missed canals to be 23%, but Costa et al. [6] and Baruwa et al. [8] found a quite small prevalence of 12%. In the present study, the overall frequency of missed canals among endodontically treated teeth was 18%, which is in between compared to the previous studies. The difference between our study and the previously reported data could be attributed to the study sample, methodology, and population; however, the results somewhat confirmed the same finding. In our study, we found the frequency of AP in RCT teeth with missed canals as 90%. Karabucak et al. [7] and Baruwa et al. [8] reported slightly lower percentage (82.8% and 82.6%, respectively), while Costa et al. [6] found a very high prevalence of 98% among teeth with missed treated canals. All studies showed a strong association between missed canals and apical periodontitis.

Maxillary molars presented with a complex RCS where the mesiobuccal (MB) root had 2 canals (the second one is known as MB2) [26–29]. The MB root has been reported to have the highest frequency of missed treated canals in many previous studies, with 85%, 65%, and 62.8% [6–8]. These findings are comparable to our finding where 61% of MB had missed treated canals, while in mandibular teeth, MB and DB canals in the first molars were the most missed treated canals in our sample (27.3% and 27.3%, respectively). This is in contrast with previously reported data on the same tooth where 62% and 62.9% of missed canals were found in the distal root [7, 8]. The difference between our finding and others might be related to the small sample size of the present study. Interestingly, in the present study, the mandibular first molar was the only RCT tooth that presented missed canals. The high rate of missed canals in this tooth might be related to the anatomic variability, both in the number of roots and root canals [30–32].

The main limitation of our cross-sectional study apart from the sample size is its nature, which evaluated the teeth at a given point of time, and the information about the situation of apical periodontitis and when the treatment is done is unknown, which means that some lesions might be healing once the treatment is just done, so a cause-effect relation cannot be established [33]. In addition, many factors could affect the treatment outcome, including techniques, materials, asepsis, preoperative status of the tooth, and expertise of the clinicians [34–36].

## 5. Conclusion

Within the limitation of the present study, the frequency of AP in RCT teeth with missed canals was high (90%), with most identified missed canals in maxillary and mandibular first molars. All measures available including CBCT and dental operative microscope should be used to maximize the identification of the RCS.

## Figures and Tables

**Table 1:** Prevalence of apical periodontitis and missed canals among endodontically treated teeth.

	Apical periodontitis		Missed treated canals	
	Yes	No	Yes	No
Maxillary teeth (*N* = 89)				
First premolars (*N* = 22)	12 (54.5)	10 (45.5)	3 (13.6)	19 (86.4)
Second premolars (*N* = 23)	18 (78.3)	5 (21.7)	1 (4.3)	22 (95.7)
First molars (*N* = 32)	25 (78.1)	7 (21.9)	13 (40.6)	19 (59.4)
Second molars (*N* = 12)	10 (83.3)	2 (16.7)	2 (16.7)	10 (83.3)
Total	65 (73.0)	24 (27.0)	19 (21.3)	70 (78.7)

Mandibular teeth (*N* = 76)				
First premolars (*N* = 2)	1 (50.0)	1 (50.0)	0 (0.0)	2 (100.0)
Second premolars (*N* = 13)	6 (46.2)	7 (53.8)	0 (0.0)	13 (100.0)
First molars (*N* = 44)	39 (88.6)	5 (11.4)	11 (25.0)	33 (75.0)
Second molars (*N* = 17)	12 (70.6)	5 (29.4)	0 (0.0)	17 (100.0)
Total	58 (76.3)	18 (23.7)	11 (14.5)	65 (85.5)

**Table 2:** Apical periodontitis among maxillary teeth with missed treated canals.

Teeth	Canal	Radiolucency	
		Yes	No
First premolars (*N* = 3)	Buccal	1 (33.3)	0 (0.0)
	Palatal	2 (66.7)	0 (0.0)

Second premolars (*N* = 1)	Buccal	0 (0.0)	0 (0.0)
	Palatal	0 (0.0)	1 (100.0)

First molars (*N* = 13)	Mesiobuccal	8 (61.5)	2 (15.4)
	Distobuccal	3 (23.1)	0 (0.0)
	Palatal	0 (0.0)	0 (0.0)

Second molars (*N* = 2)	Mesiobuccal	1 (50.0)	0 (0.0)
	Distobuccal	1 (50.0)	0 (0.0)
	Palatal	0 (0.0)	0 (0.0)

**Table 3:** Apical periodontitis among mandibular teeth with missed treated canals.

Teeth	Canal	Apical periodontitis	
		Yes	No
First premolars (*N* = 0)	One root	—	—

Second premolars (*N* = 0)	One root	—	—

First molars (*N* = 11)	Mesiobuccal	3 (27.3)	0 (0.0)
	Mesiolingual	2 (18.2)	0 (0.0)
	Distobuccal	3 (27.3)	0 (0.0)
	Distolingual	2 (18.2)	0 (0.0)
	Mesial	0 (0.0)	0 (0.0)
	Distal	1 (9.0)	0 (0.0)

Second molars (*N* = 0)	Mesiobuccal	—	—
	Mesiolingual	—	—
	Distobuccal	—	—
	Distolingual	—	—
	Mesial	—	—
	Distal	—	—

## Data Availability

The data supporting the findings of this study are available from the corresponding author upon reasonable request.
